# A School Doctor on the General Practitioner

**Published:** 1912-07-27

**Authors:** 


### STAFFING THE SCHOOL CLINIC.

### A School Doctor on the General Practitioner.

BY ONE OF THE FORMER WHO IS NOT ONE OF THE LATTER.

A great deal of discussion has been going on of late with reference to the relation of the general practitioner to the medical inspection of school children in general, and to the school clinic in particular. Following the lead given by provincial branches, the British Medical Association has declared itself strongly in favour of the general practitioner undertaking not only the actual inspection of the children, but the management of the clinic as well. It is argued that the general practitioner is an all-round man: that he is as capable of removing tonsils and adenoids and doing the ordinary minor operations that are likely to be performed in a school clinic as any special school doctor who, it is alleged, is usually not a specialist, but merely a clinical assistant at some hospital; and that he is the only fit and proper person to undertake the duties both of examining the children at school and treating their ailments at school clinics.

This attitude of mind towards the general practitioner is a very comforting one. Unfortunately it cannot be persisted in by anyone who has had any experience of the manner in which the medical inspection of schools is carried out by the general practitioner, or the way in which children are treated at school clinics staffed by general practitioners. There are one or two such clinics near London, and we recommend anyone who is able to judge for himself to visit these and to see what is done and in what manner of way it is being done. A school doctor who has had some experience of the results achieved by one of these—and perhaps the best known of these clinics—is very emphatic on some points of mismanagement.

### Examples of Mismanagement.

The worst fault lies in the slovenliness and want of thoroughness in treatment. Spectacles are ordered which are not suitable; and this even in cases where mydriatics are used. In such cases there is usually a very slight degree of astigmatism, which needs very careful attention. . . . Again, the operation for adenoids and tonsils is very carelessly done. In 15 per cent. of cases I have had to recommend that the operation should be repeated, and have advised the parents to take the children to some reputable children's hospital, as I could not honestly recommend the clinic. . . . Discharging ears are treated in a fashion that would be amusing were it not that the results are so pitiable. . . . As for orthopædic cases, the essentials of treatment appear to be unknown. . . . Medical cases, on the whole, are more satisfactory, but I have seen several children with bronchiectasis labelled as suffering from bad phthisis, and others, who had nothing the matter with them, and who had been sent to the clinic for anæmia, diagnosed as "bad heart disease." From my experience of this clinic I consider it inexpedient that the management of school clinics should be entrusted wholly to general practitioners. If this clinic had the benefit of a few specialist consultants, its results would be much better than they are at present.

Another school doctor, who is an eye specialist, is equally emphatic. He says:—

Eye work is almost never a success when carried out by the general practitioner. He invariably underestimates its importance, and thinks that after being initiated into the mysteries of "refractions" he can do anything. As everyone who has really worked at this subject knows, ophthalmology is a very difficult subject, and no part of it demands greater care and skill than refraction work. . . . I have had considerable experience of the work done at —— clinic, and I cannot say that I am at all satisfied with it. Where the errors are easy of correction, the spectacles ordered usually answer; but where there is any difficulty in estimating, the results are usually quite unsatisfactory.

### The Case Against the "G. P."

These are unprejudiced opinions given by men who are not connected with any clinic but who, engaged in daily routine duties of school inspection, see a good deal of the results of the work carried on at such centres of treatment. The argument against the general practitioner staffing the school clinic was considered at some length by Dr. Priestley, the School Medical Officer for Staffordshire, in a paper read before the last annual meeting of the British Medical Association. Dr. Priestley favours the scheme in which the clinic is managed by the school doctor. *School Hygiene*, in commenting upon this opinion, remarks as follows:—

We agree with Dr. Wynne that "the general practitioner is a far more competent person for the purpose (of treatment) than a young person just turned out of the hospital or public health laboratory, who was supposed to be a specialist in eye, ear, and throat work, and mental diseases at the age of twenty-four." Specialists are not required to deal with these diseases; they require men with some clinical experience, men who have got the ordinary skill of the average general practitioner (who has not got rusty); men who will deal with children and their parents as with human beings . . . . who will, in short, be as civil and kind to those attending the clinic as he would be to his well-to-do persons.

The bad grammar of this comment is on a par with its faulty reasoning. No one proposes to staff school clinics with persons just turned out of hospital or laboratory (whatever that may mean, though we take it that Dr. Wynne implies practitioners who have just qualified). The age of the individual does not in the least matter; we have experience of a young clinical assistant diagnosing and treating cases in a manner that compared very favourably with that of a practitioner old enough to be his grandfather, and to those who use the argument of extreme youth we can only recommend the attentive study of Pitt's famous reply. The contention that diseases of the nose and throat, eye and ear are not specialties in the best sense of the term is one which is contrary to the whole trend of modern medical opinion. A general practitioner who has not studied these subjects as specialties is *not* as competent to treat these diseases as a clinical assistant who has conscientiously worked in a special department. To be able to treat these conditions, the practitioner must not only not be rusty but he must devote a certain amount of time every day to the study of new methods and to becoming acquainted with the most recent literature. A general practitioner finds no time to do this, unless he takes up a specialty in earnest.

A clinical assistant, who in the absence of his chief is in charge of his special department, undertakes this study as a matter of routine; it is in his interest to do so, for he is naturally anxious to make himself proficient in the specialty which he has selected. The last part of the editorial opinion above quoted is less a contention than an innuendo. It implies, if it implies anything, that the specialist will not treat his patients at the clinic as human beings, and that the general practitioner will. While it is an undoubted fact, and one which has been often alluded to, that children sent from schools to the out-patient departments of general or special hospitals are often very badly attended to, it is an equally indubitable fact that the general practitioner does not treat his club patients as he would his more well-to-do patients. The general practitioner, therefore, cannot honestly admonish the specialist unless he first reforms his own methods. But it is bad logic, and in this matter, bad charity, to argue from the particular to the general. The specialist or general practitioner attached to a school clinic takes his appointment seriously in the majority of cases, and we give both of them credit for conscientiousness and common honesty in endeavouring to do their best. The implied accusation against the former is as uncalled for as the often made charge that the general practitioner opposes the establishment of school clinics because he thinks that it will curtail his practice.

The main argument against the employment of the general practitioner in the school clinic is the one which *School Hygiene* itself admits when it remarks that the general practitioner should not be left in uncontrolled charge. We prefer to see these clinics staffed by whole or part time school doctors, who are assisted by honorary or paid consulting specialists, not for purposes of control but for purposes of consultation. The general practitioner cannot concentrate himself on the special work which such clinics entail, and he would be the last person in the world to suggest that he should be a whole-time official under the control of the education authorities.

